# Hyperhomocysteinemia in bilateral anterior ischemic optic neuropathy after conventional coronary artery bypass graft: a case report

**DOI:** 10.1186/s13256-017-1539-1

**Published:** 2018-01-17

**Authors:** A. Niro, G. Sborgia, A. Sborgia, G. Alessio

**Affiliations:** 1 Department of Medical Sciences, Neuroscience and Sense Organs,, University of Bari “A. Moro”, Eye Clinic, Piazza G. Cesare, 11, 70124 Bari, Italy; 2 Clinica Oculistica Azienda Ospedaliero-Universitaria Policlinico Bari, Piazza G. Cesare,11, 70124 Bari, Italy

**Keywords:** Hyperhomocysteinemia, Anterior ischemic optic neuropathy, Conventional coronary artery bypass graft (CCABG)

## Abstract

**Background:**

The incidence of anterior ischemic optic neuropathy after coronary artery bypass graft procedures ranges from 1.3 to 0.25%. The mechanisms of anterior ischemic optic neuropathy after cardiovascular procedures remain undefined but many systemic and related-to-surgery risk factors could underlie anterior ischemic optic neuropathy. In this case, we report a rare presentation of a bilateral anterior ischemic optic neuropathy after coronary artery bypass graft and speculate on the preoperative hyperhomocysteinemia as an independent risk factor for anterior ischemic optic neuropathy.

**Case presentation:**

A 56-year-old white man, a tobacco smoker with type 2 diabetes and coronary artery disease, underwent a conventional coronary artery bypass graft with extracorporeal circulation. In spite of ongoing anti-aggregation, antithrombotic, and vasodilator therapy, 10 days after the surgery he complained of severe bilateral visual loss. Funduscopy and fluorescein angiography revealed a bilateral anterior ischemic optic neuropathy. Analysis of preoperative laboratory tests revealed hyperhomocysteinemia.

**Conclusion:**

Hyperhomocysteinemia could increase the risk of ocular vascular damage and bilateral ocular involvement in patients who have undergone conventional coronary artery bypass graft.

## Background

Vision loss after non-ocular surgery is a rare event. Its incidence is 0.002% for all surgery treatments [1, 2]. The most common neuro-ophthalmologic causes of visual impairment after non-ocular surgery are the ischemic optic neuropathies (ION): anterior ION (AION) or posterior ION (PION). After coronary artery bypass graft (CABG) procedures the incidence of ION ranges from 0.11 [3] to 0.06% [4, 5]. The incidence of AION after CABG procedures ranges from 1.3 to 0.25% [3, 6]. CABG is a vascular bypass implantation at the site of narrowing or blockage of coronary arteries. Healthy arteries or veins are grafted to the coronary arteries to allow the reperfusion of the ischemic area of the heart. During a conventional CABG (CCABG) procedure, extracorporeal circulation (ECC) is used. The mechanisms of visual loss after CCABG remain undefined but hypotension, anemia, and other factors could underlie the AION [7]. Hyperhomocysteinemia, in addition to being an independent risk factor for vascular diseases and myocardial infarction [8], is a risk factor for AION [9, 10]. Weger *et al.* believe that determination of homocysteine level might have a diagnostic value in patients with AION [11]. The homocysteine level could be routinely measured before CCABG procedure.

## Case presentation

A 56-year-old white man, a tobacco smoker with type 2 diabetes and coronary artery disease, underwent cardiac revascularization. During CCABG, his internal mammary artery as arterial graft and double bypass with saphenous vein were used. He was under therapy with enoxaparin sodium 6000 I.U. anti-Xa activity (aXa) twice a day, acetylsalicylic acid 100 mg daily, prednisone 5 mg daily, and mild diuretic therapy. Ten days after cardiac surgery he complained of bilateral visual loss: best corrected visual acuity (BCVA) was 0.9 LogMAR in right eye (RE) and 1.0 LogMAR in left eye (LE). Afferent pupillary defect (APD) was revealed in his LE. In both eyes computerized perimetry showed an absolute and general reduction of the retinal sensitivity within 30 degrees around the fixation point.

**Fig. 1:**
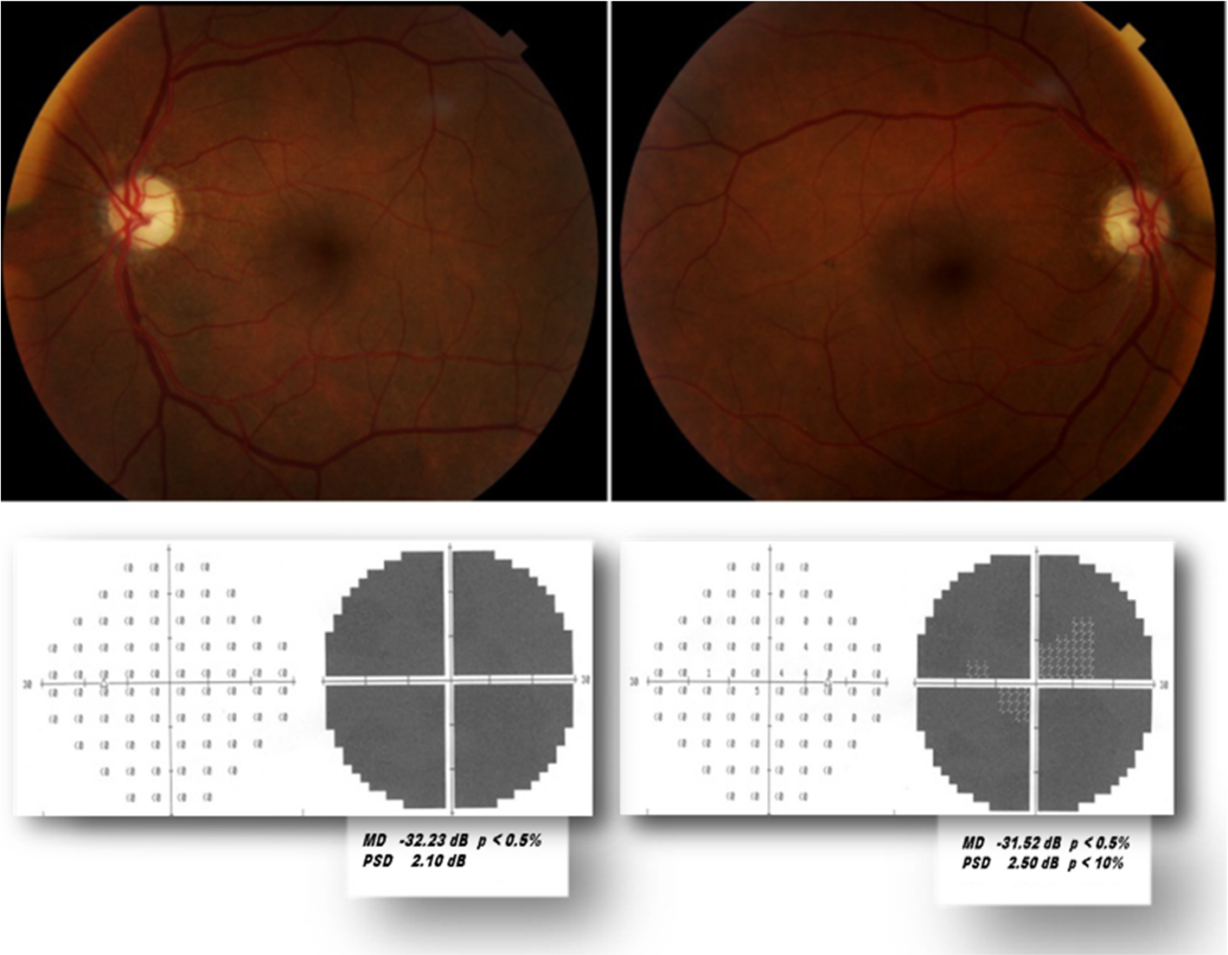
(*first row*) Fundus photograph of both eyes showing bilateral temporal pallor of the optic disc; (*second row*) 8 weeks after the anterior ischemic optic neuropathies occurrence, visual field test of both eyes showing a complete defect with a very low mean defect value

In both eyes the high intraocular pressure (IOP) (26 mmHg) was successfully medically managed. We observed on funduscopy bilateral pallid optic disc edema and splinter hemorrhages at the optic disc edge. In both eyes fluorescein angiography showed hypofluorescence of the optic disc in the early phases due to filling delay followed by hyperfluorescence with leakage from disc capillaries in the late phases of the angiogram. A neuroimaging study revealed no signs of intraorbital pathology, elevated intracranial pressure, or hemorrhages. From these findings we diagnosed AION. The analysis of preoperative laboratory tests revealed a mild anemia (hematocrit, 38%; hemoglobin, 11.5 g/dL) and a high blood level of homocysteine (19.8 μmol/L). When AION was diagnosed, we prescribed folic acid and B-complex vitamins to our patient. During the hospitalization, his visual acuity reduced further: BCVA was 1.0 LogMAR in RE, hand motion in LE. After 8 weeks, funduscopy revealed bilateral temporal optic disc pallor without edema and a total visual field defect in both his eyes (Fig. 1).

## Discussion

In a review of 5.6 million patients from National Inpatient Sample the visual loss ratio after cardiac surgery was 8.64/10,000 [12]. AION occurs most often after cardiac surgery [13]. AION is the result of an insufficient blood flow provided by short posterior ciliary arteries to the retrolaminar portion of the optic nerve head.

In a previous study, 17 patients (0.06%) out of a total of 27,915 who underwent CABG had perioperative ION [4]. Kalyani *et al.* found 11 patients (0.113%) with perioperative optic neuropathy out of 9701 surgical patients requiring cardiopulmonary bypass [5]. In a case-control analysis of 126,666 surgical procedures the authors identified 17 patients (0.013%) with perioperative ION [14]. Shapira* et al.* found the development of AION in 8 patients (1.3%) out of 602 consecutive cardiac surgery patients [3].

In CABG there are many correlated-to-surgery risk factors able to influence ocular blood flow causing AION: hypothermia induced during surgery could increase blood viscosity, leading to watershed infarction of the optic nerve [15] and lowering cerebral and ocular blood flow [16]; the activation of complement cascade with increase of C3a acts as a smooth-muscle spasmogen [17]; the long duration of the CABG procedure is associated with greater inflammation and higher levels of endogenous catecholamines that may act synergistically to produce vasoconstriction and ischemia in the posterior ciliary circulation [3, 4]; a lower postoperative hemoglobin value (≤8.5 g/dL) results in a reduction in oxygen-carrying capacity and subsequent ischemia [4]; a coronary angiogram within 48 hours of surgery acts as a risk factor by an unclear mechanism [4]; an arrhythmia could induce a reduction in cardiac output [5]; the large fluid infusion to support blood pressure [4] reduces oxygen-carrying capacity by hemodilution; the inotropic medications [4]; a blood loss associated or not with arterial hypotension causes activation of the sympathetic nervous system followed by vasoconstriction which induces choroidal and optic nerve ischemia [18]; and the use of vasoconstricting agents to correct intraoperative hypotension has also been suggested to promote optic nerve ischemia [15, 18, 19].

However, in a previous case-control analysis of 126,666 surgical procedures the authors concluded that perioperative ION can occur in the absence of any unusual or atypical fluctuations in many hemodynamic variables during the perioperative period [14]. In that paper there was no difference between 17 patients with perioperative ION and 34 control patients in terms of preoperative variables like age, medical history, body mass index, mean arterial pressure, and hematocrit. We know that there are predisposing ocular risk factors of AION as preexisting “disc-at-risk” configuration, but the presence of this configuration cannot be determined in a swollen or atrophic optic disc, and a vascular disorder of the optic nerve was not noted in the clinical history of our patient.

Furthermore, there are many systemic, non-correlated-to-surgery risk factors of AION like high serum cholesterol, triglycerides, hyperlipidemia, hyperfibrinogenemia, prolonged tobacco smoking history, hypertension, anemia, and diabetes mellitus [20].

In our patient we found different systemic and non-correlated-to-surgery risk factors like hypertension, mild anemia, and diabetes which may reduce tolerance to hypotension of optic nerve blood flow during CABG procedure [21, 22]. Different authors [23–26] found postoperative anemia to be a predisposing cause for AION. Mansour *et al.* reported that severe anemia in patients undergoing CABG appears to be a risk factor for AION, especially in diabetics, and needs prompt correction to prevent or reverse ischemic ocular events [27]. However, in our case the hematocrit and hemoglobin values were much higher than that reported in previous papers.

Furthermore, we found a moderate hyperhomocysteinemia probably correlated with our patient’s tobacco smoking history. Hyperhomocysteinemia is observed in approximately 5% of the general population and is associated with an increased risk of many disorders, including vascular and neurodegenerative diseases, autoimmune disorders, diabetes, renal diseases, neuropsychiatric disorders, and cancer [28]. Hyperhomocysteinemia is an independent risk factor for atherosclerosis of the graft, one of the main limitations of long-term survival of patients who have undergone CABG [29]. Furthermore, homocysteine is known as a risk factor for vascular eye pathologies [11]. Hyperhomocysteinemia acting as an oxygen free radical causes dysfunction and necrosis of endothelial cells, and proliferation of smooth muscle cells with subsequent fibrosis and calcification of the vessel [30]. On the other hand, high levels of homocysteine induce the formation of atheroma (atherosclerotic plaque) and the proliferation of smooth muscle cells resulting in endothelial damage and reduced elasticity of the vessel [30]. Hyperhomocysteinemia also increases platelet adhesiveness and aggregation [30]. We have to report that in this case the preoperative and postoperative antiplatelet and anticoagulant therapy did not avoid the optic nerve ischemia.

As reported in our case, a moderate increase of IOP after surgery could occur [31] due in part to hemodilution with a decrease in colloidal osmotic pressure induced by bypass pump circulation [32].

The CCABG procedure mentioned in this case report used an extracorporeal circulation with cardiopulmonary bypass which may lead to many postoperative complications like AION.

## Conclusions

Several preoperative and intraoperative clinical conditions increase the risk of ocular complications like AION after CCABG procedure; so, preoperative hyperhomocysteinemia could increase the risk of ocular vascular damage and bilateral ocular involvement. We would like to conclude with the hypothesis that it would be worthwhile to investigate the potential benefit of treating preexisting hyperhomocysteinemia in patients undergoing CABG.
